# Population structure of the NPGS Senegalese sorghum collection and its evaluation to identify new disease resistant genes

**DOI:** 10.1371/journal.pone.0191877

**Published:** 2018-02-14

**Authors:** Hugo E. Cuevas, Louis K. Prom, Giseiry Rosa-Valentin

**Affiliations:** 1 USDA-ARS, Tropical Agriculture Research Station, Mayaguez, Puerto Rico; 2 USDA-ARS, Southern Plains Agriculture Research Center, College Station, Texas, United States of America; National Bureau of Plant Genetic Resources, Pusa India, INDIA

## Abstract

Sorghum germplasm from West and Central Africa is cultivated in rainy and high humidity regions and is an important source of resistance genes to fungal diseases. Mold and anthracnose are two important biotic constraints to sorghum production in wet areas worldwide. Here, 158 National Plant Germplasm System (NPGS) accessions from Senegal were evaluated for agronomic traits, anthracnose, and grain mold resistance at two locations, and genetically characterized according to 20 simple sequence repeat markers. A total of 221 alleles were amplified with an average of 11 alleles per locus. Each accession had a unique genetic profile (i.e., no duplicates), and the average genetic distance between accessions was 0.42. Population structure and cluster analysis separated the collection into four populations with pairwise F_ST_ values >0.15. Three of the populations were composed of Guinea-race sorghum germplasm, and one included multiple races. Anthracnose resistant accessions were present at high frequency and evenly distributed among the three Guinea-race populations. Fourteen accessions showed resistance to grain mold, and eight were resistant to both diseases. These results indicated that the NPGS of Senegal is a genetically diverse collection with a high frequency of disease resistant accessions. Nevertheless, its population structure suggests the presence of few sources of resistance to both grain mold and anthracnose, which are fixed in the germplasm. The phenotypic and genotypic information for these accessions provides a valuable resource for its correct use to broaden the genetic base of breeding programs.

## Background

Sorghum [*Sorghum bicolor* (L.) Moench] is the fifth most important cereal after maize, wheat, rice, and barley [1], and is becoming an important source of grain and cellulosic-based ethanol. This highly diverse crop is the result of multiple re-domestication processes that led to the development of five different races (Bicolor, Durra, Guinea, Caudatum, and Kaffir) characterized by their inflorescence type [2]. These races are specific to different regions of Africa and are associated with particular environments [3, 4]. The recombination of sorghum cultivars and their selection and ongoing evolution in an array of environments resulted in a highly phenotypically and genetically diverse crop.

*Ex-situ* germplasm collections (i.e., gene banks) are an important genetic resource for breeding programs and geneticists worldwide. In fact, these germplasm collections provide genetic variation for crop improvement. Hence, *ex-situ* germplasm collections must be managed properly to enable breeders to continue to develop improved cultivars [5]. Multiple sorghum germplasm collections are presently maintained by different countries. The largest worldwide sorghum collection is maintained by the National Plant Germplasm System (NPGS) of the United States Department of Agriculture (USDA) which includes >41,860 accessions from 114 countries. To use these large resource adequately, breeding programs must understand the variation within the germplasm collection [6]. The use of molecular markers to analyze the genetic diversity and population structure of germplasm collections provides the information needed to improve phenotyping and conservation. Recently, large-scale genetic characterizations of germplasm collection have improved our understanding of the domestication, diversification, and dispersion of sorghum worldwide [7–10]. However, genetic characterization of germplasm from particular countries or geographical regions is necessary to create allele mining programs that comprehensively evaluate the genetic diversity. For instance, the GBS analysis of 354 NPGS Ethiopian accessions identified >150,000 SNPs and revealed abundant genetic diversity, identifying 11 different populations [11]. The genomic characterization of particular NPGS collections provides the knowledge to optimize experimental designs based on population structure and genetic diversity.

Sorghum germplasm from West and Central Africa is well adapted to the wet environments characteristic of these regions [10], and is therefore considered an important source of resistance genes to fungal diseases. Grain mold and anthracnose are two important biotic constraints to sorghum production in rainy and humid regions worldwide [12, 13]. Grain mold is a disease caused by pathogenic and opportunistic fungi, including various *Fusarium* species (such as *F*. *thapsinum* Klittich, Leslie, Nelson, & Manasas; *F*. *semitectum* Berk & Ravenel; *F*. *proliferatum* (Matsushima) Nirenberg; and *F*. *andiyazi* Marasas, Raheeder, Lamprecht, Zeller, & Leslie) and *Curvularia lunata* (Wakk.) Boedijn and *Alternaria alternata* (Fr.) Keissler, which are the most prevalent species worldwide [14, 15]. Yield losses can reach up to 100% in highly susceptible cultivars [16], and the most effective method for its control is the use of resistant cultivars [17]. Despite the recent identification of new sources of resistance in germplasm from Sudan [18], Burkina Faso [19], Uganda [20], and other West and Central African countries [21], additional sources are necessary to develop new resistant varieties.

Anthracnose is caused by the fungus *Colletotrichum sublineola* P. Henn, in Kabát & Bubák [syn. *C*. *graminicola* (Ces.) G. W. Wilson], and is one of the most destructive diseases because it infects all aerial tissues of the plant [13]. The pathogen population is highly diverse genetically, making it difficult to obtain a widespread or durable source of resistance [22]. The deployment of multiple resistance genes is therefore necessary to control the disease. Recent evaluation of germplasm from China, Ethiopia, Mali, Mozambique, Botswana, Malian, Sudan, Uganda, and Zimbabwe identified resistant lines [19, 20, 23–27]; however, the lack of knowledge about genetic relationships between resistant germplasm and its mode of inheritance limits the use of these resources in breeding programs. In this regard, the identification and selection of the most genetically diverse and exotic germplasm for inheritance studies is the first step towards its introgression into breeding programs.

In the current study, 158 NPGS Senegal accessions were evaluated for anthracnose and grain mold resistance and genetically characterize with 20 simple sequence repeats (SSR) to: 1) determine population structure of NPGS Senegal collection, 2) identify resistance accessions to both diseases, and 3) determine the possible number of new resistance sources for breeding programs based on the population structure analysis.

## Materials and methods

### Germplasm

A total of 158 Senegalese sorghum accessions in the current NPGS collection (i.e. 44% of the collection) were selected according to collection site information contained in the Germplasm Resources Information Network database. This core set included 115 different collection sites representing the eco-geographical variation of Senegal.

In addition, the sorghum breeding lines BTx623, RTx430, Sureño, and RTx2911, the converted tropical lines SC748-5 and SC112-14, and the grain mold resistant accession PI 267548 from Sudan [18] were included in the phenotypic analysis as references.

### Population genetic analysis

#### DNA isolation and SSR analysis

Genomic DNA was isolated from seedlings (2–3 weeks old) of the 158 Senegal accessions based on the methods of Guillemaut and Marechal-Drouard [28], quantified with a nanophotometer (IMPLEN, CA, USA), and normalized to 25 ng/μL for polymerase chain reaction. Twenty genomic sorghum SSR markers were strategically selected based on their high polymorphic information content (PIC) value, as determined in the analysis of eight Senegal accessions in the ICRISAT mini-core reference set [29]. Forward SSR primers were appended at the 5′ end with the M13 sequence to obtain a fluorescent PCR product for capillary electrophoresis [30]. Two fluorescent amplified PCR products were multiplexed according to amplicon size, mixed with 0.5 μL of carboxy-X-rhodamine (ROX) standard (CHIMERx, Milwaukee, WI, USA) and 10 μL of Hi-Di formamide (Applied Biosystems, Foster City, CA, USA), and run through an ABI *3730xl* genetic analyzer (Plant Genome Mapping Laboratory, Athens, GA, USA). Fragment analysis and allele scores were determined by GeneMarker Software version 2.2 (SoftGenetics, State College, PA, USA).

#### Population structure and genetic diversity analysis

Population structure and admixed ancestry were assessed using a model-based clustering method implemented in STRUCTURE 2.1 [31]. The analysis was performed using an admixture model with correlated allele frequencies, 50,000 burn-in periods, 20,000 Monte Carlo Markov Chain (MCMC) replicates, and three independent runs for each *k* value set from 1 to 12 [7]. The actual number of populations was determined using the ad hoc statistic Δ*k* based on the rate of change in the log probability of data between successive *k* values [32], as implemented by Structure Harvester software [33]. Ten additional independent runs for the actual number of populations (50,000 burn-in periods and 300,000 MCMC replicates) were matched by permutation in CLUMPP [34] to generate the optimum alignment over multiple runs. Accessions with an ancestry membership coefficient <0.70 were considered admixed.

Overall and each population’s genetic diversity was estimated based on the total number of alleles, allelic richness, observed and expected heterozygosity, and fixation index of the markers. The pairwise fixation index (F_ST_) between populations was estimated based on the method of [35] using the R package Hierfstat [36].

The pairwise genetic distance between the 158 Senegalese accessions was calculated based on allele share distance, as implemented in GeneAlex [37] using the 221 identified alleles. The resulting matrix was subjected to clustering analysis using the neighbor-joining method and visualized in Interactive Tree of Life [38].

### Phenotype characterization

#### Phenotypic analysis

The 158 Senegalese accessions and seven references were evaluated at Isabela and Mayaguez, Puerto Rico, during the short day-length season of 2014–2015. In both locations, a randomized complete block design consisting of two blocks with plots of 1.8 m in length and 0.9 m between rows was used. Plants were maintained using standard management practices, and weeds were controlled by mechanical tillage and hand hoeing.

Flowering time (FL), plant height (PH), panicle length (PL), panicle diameter (PD), and the ratio of PL/PD were obtained for each accession/plot at both locations. FL was defined as the number of days until 50% of the plants within a plot reached anthesis. PH referred to the average distance from the base of the main stalk (i.e., soil) to the top of the panicle at maturity for two representative plants per accession/plot. PL referred to the distance from the first rachis to the top of the panicle, and PD to the widest section of the panicle [39].

The data from all locations were combined and subjected to analysis of variance using the *Proc mixed covtest* method *type 3* procedure of SAS (SAS Institute, Cary, NC, USA). The location and accession were considered fixed, whereas blocks were treated as random effects. The performances of the accessions and Senegal populations were estimated based on least square means and compared using the Tukey-Kramer honest significant difference test.

#### Anthracnose response

The inoculation and disease assessment methods used were similar to those described by Prom et al. (2009). Two fungal cultures were prepared with different isolates of *C*. *sublineolum* representing the pathotypes present at the Isabela and Mayaguez experimental farm. The five pathotypes of Isabela were previously characterized [22], whereas the three pathotypes from Mayaguez have not been characterized. These isolates were used to colonize sorghum seeds, which were later placed into the sorghum leaf whorls 30–40 days after planting. Disease assessment was performed before harvesting using a scale of 1–5 as follows: 1 = no symptoms or chlorotic flecks on leaves; 2 = hypersensitive reaction on inoculated leaves but no acervuli in the center; 3 = lesions on inoculated leaves with acervuli; 4 = necrotic lesions with acervuli observed on inoculated and bottom leaves with infection spreading to middle leaves; 5 = most leaves are dead due to infection including infection on the flag leaf. This rating system was used to categorize accessions into resistant (scores of 1 and 2) and susceptible cultivars (scores of 3–5). The Chi square test was used to determine whether the frequency of resistant accessions in one population was higher/lower than that expected based on the frequencies of the collection.

#### Grain mold

The inoculation and disease assessment methods used were similar to those described by Prom, Isakeit (20). The grain mold fungus used was previously isolated and characterized based on conidium, conidiophore, and colony morphology and color from infected seeds of Isabela, P.R. [40], and named CL-Isa-1, FT-Isa-1, and FV-Isa-1 for *C*. *lunata*, *F*. *thapsinum*, and *F*. *verticillioides*, respectively. These pathotypes were cultured on ½ strength potato-dextrose agar (PDA) at 25°C for 7 days. The conidial mixtures were prepared according to Prom, Isakeit (20), combining approximately 50 mL of each conidial suspension of *C*. *lunata*, *F*. *thapsinum*, and *F*. *verticillioides* into a 1.9 L spray bottle filled with distilled water. Although it was not possible to deliver equal conidial concentrations each day or the same number of spores for the different fungi, the mixture ensured the presence of multiple fungal spores during anthesis. These conidial mixtures were thoroughly agitated before inoculation, and, because of differences in developmental stages, panicles were inoculated on different dates. Three panicles evenly distributed within the plot (i.e., one plant from the start, middle, and end of the plot) with uniform flowering time were inoculated with the conidial mixtures daily from the first to the last anthesis day. Panicles were covered with a mesh harvest bag (MIDCO, Kirkwood, MO, USA) to avoid bird damage and ensure grain exposure to weathering effects; panicles were hand harvested at 35–40 days after anthesis, dried, and threshed using a single plant thresher (Almaco Single Plant and Head Thresher, Allan Machine Company, Nevada, IA, USA).

Grain mold resistance response was determined based on seed visual rating and germination rate [20]. Approximately 400 seeds from each treated panicle were assessed for grain mold severity using a scale of 1–5 [17, 41] as follows: 1 = seed is bright with no mold and no discoloration due to weathering (highly resistant); 2 = seed is not as bright and has little or no mold, but has some discoloration [1–10% molded kernels (resistant)]; 3 = seed is not bright, and there is some mold and discoloration [11–25% molded kernels (moderately resistant)]; 4 = seed is almost entirely covered in mold and is deteriorating [26–50% molded kernels (susceptible)]; 5 = seed is covered entirely with mold, is deteriorated, and looks dead [>50% molded kernels (highly susceptible)]. Germination rate was determined according to the number of seeds that germinated after 10 days from 30 seeds from each treated panicle (i.e., 90 seeds from each experimental unit). The seeds were planted in flats containing Metro Mix 200 potting medium, and incubated in the greenhouse at room temperature. The performance of accessions was estimated based on least square means and compared with that of grain mold resistant accession PI 267548 [18] using the Tukey-Kramer honest significant difference test.

#### Environmental conditions

Data on the environmental conditions during both experiments were obtained from the USDA- Natural Resource and Conservation Soil (NRCS) weather station located in the TARS experimental farm at Isabela (http://www.wcc.nrcs.usda.gov/nwcc/site?sitenum=2052) and Mayaguez (http://wcc.sc.egov.usda.gov/nwcc/site?sitenum=2112), Puerto Rico. Daily minimum, maximum, and average temperature and relative humidity values were collected during both experiments (Appendix 1). The relative humidity and temperature were 90.3% and 24.4°C in Isabela, P.R., and 83.6% and 24.7°C in Mayaguez, P.R. Aerial watering (Raingun Irrigation) was supplied in the absence of rainfall to increase relative humidity during both experiments.

## Results

**Fig 1:**
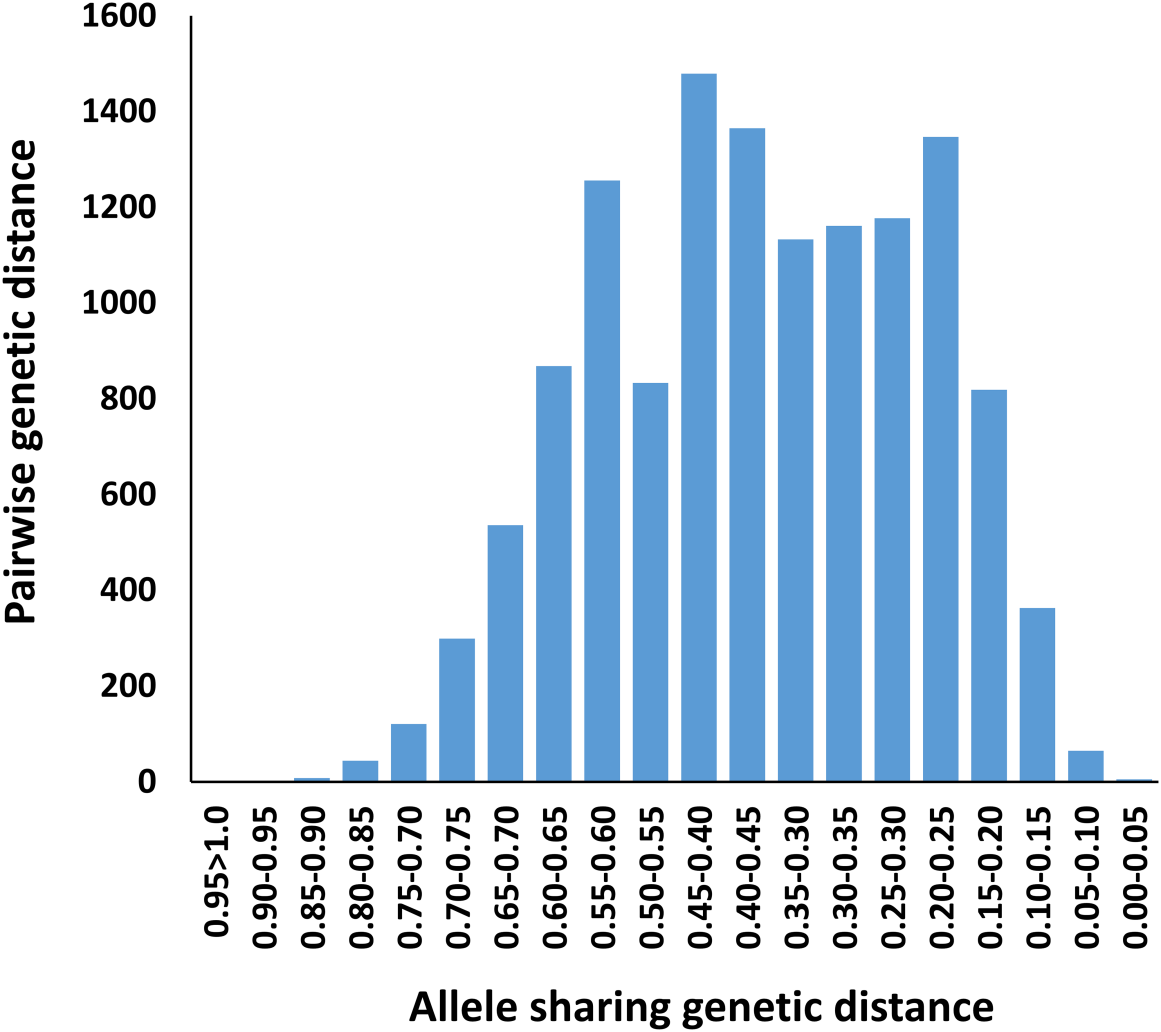
Distribution of allele sharing genetic distance among 158 NPGS Senegal sorghum accessions.

The core set evaluated in the present study represented 44% of the Senegal collection, including eight sorghum races distributed into Guinea (124), Guinea-Bicolor (4), Guinea-Caudatum (3), Durra (6), Durra-bicolor (2), Durra-Caudatum (1), Caudatum (1), and Kaffir-Caudatum, and 16 accessions without race classification. The genetic profile of each accession was determined by the combination of alleles for the 20 SSR loci tested, and used to determine population structure, allelic richness, and the presence of duplicate germplasm in the collection. A total of 221 alleles were amplified from the 20 SSR loci with an average of 11 alleles per locus. No genetic profile was duplicated, and the average genetic distance between accessions was 0.42 (Fig 1). Of the 12,880 pairwise genetic distances, only one was larger than 0.90 (PI 514299 vs. PI 514301). The average observed heterozygosity (0.25) was greater than that reported in previous studies with sorghum landraces from the NPGS collection [6, 23, 42]. However, this could be attributed to the fact that the SSRs were selected based on their previously reported high PIC value among Senegalese germplasm collections [29]. These results indicate that the NPGS Senegalese germplasm collection is highly genetically diverse, and it has been adequately preserved for further use in sorghum breeding programs.

### Population structure

**Fig 2:**
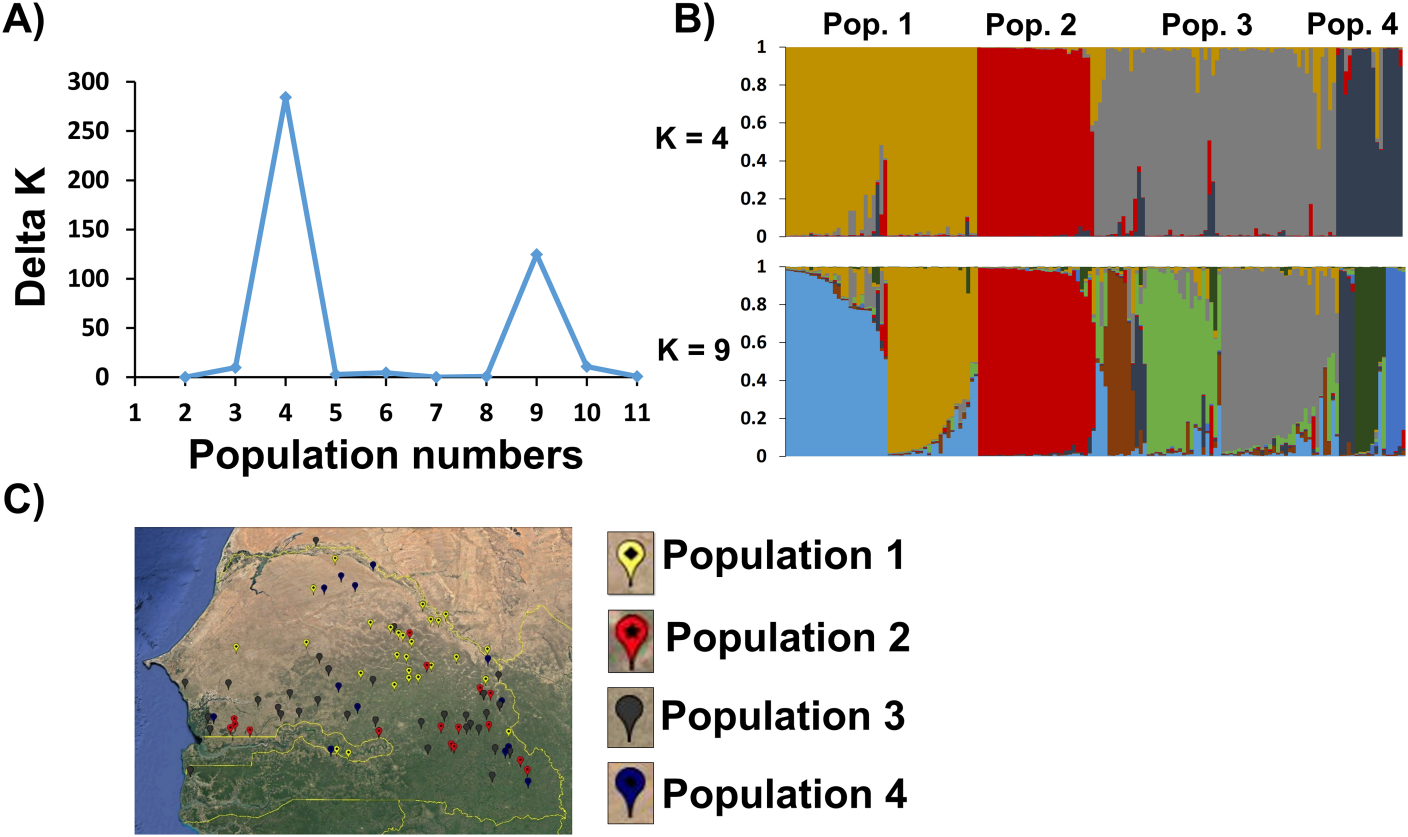
Population structure analysis of 158 NPGS Senegalese sorghum accessions. (A) The optimal number of populations was four based on the delta K method. (B) Hierarchical organization of the genetic relationships of the 158 Senegal accessions. (C) Collection sites of 107 Senegal accessions.

Defining the population structure of the NPGS Senegal collection is necessary to preserve and harness its genetic diversity. Based on the delta of ad hoc posterior probability [32] from STRUCTURE, we identified four ancestral populations (referred to hereafter as populations 1, 2, 3, and 4) comprising 147 accessions (91%) (Fig 2). Indeed, the F_ST_ between these populations ranged from 0.14 (population 1 vs. population 3) to 0.42 (population 1 vs. population 2), indicating a relatively high level of genetic differentiation (Table 1). Remarkably, populations 1, 3, and 4 could be disrupted into 3, 3, and 2 subpopulations (Fig 2), respectively. Moreover, accessions classified into the Guinea-race, domesticated in the West Africa region [43], were dispersed only in populations 1, 2, and 3, indicating that the observed population structure most likely evolved from selection and adaptation to specific Senegalese environments. By contrast, population 4 comprised multiple sorghum races that could represent recent introgression from neighboring countries. Although the collection site of the majority of these accessions is known, it is not precise because they were collected more than 70 years ago and represent multiple independent germplasm collections. Therefore, the population structure could not be associated with the eco-geographical regions of Senegal (Fig 2).

**Table 1:** Pairwise estimates of F_ST_ in NPGS Senegal sorghum populations based on the analysis of 221 SSR alleles.

	**Population 1**	**Population 2**	**Population 3**
**Population 2**	0.44		
**Population 3**	0.15	0.33	
**Population 4**	0.24	0.31	0.19

**Fig 3:**
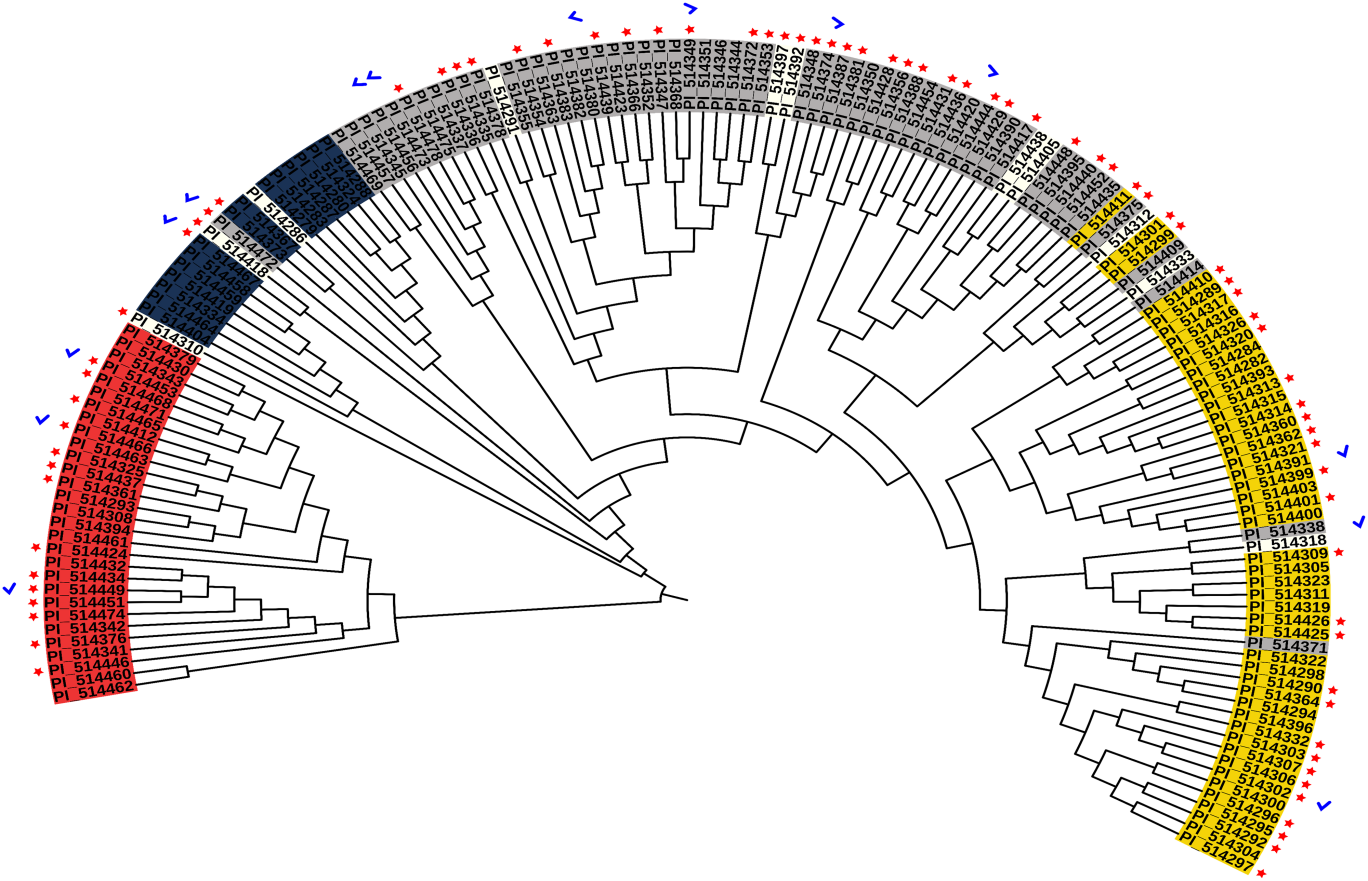
Neighbor-joining tree of 158 NPGS Senegalese accessions. Admixture accessions are not colored. Red stars and blue check marks refer to anthracnose and grain mold resistant accessions, respectively.

The unrooted neighbor-joining tree was used to understand the genetic relationships between these populations. The results of this analysis were consistent with the inferred population structure (Fig 3), with the largest clade including populations 1, 3, and 4, whereas population 2 was placed in a single clade. Indeed, the highest pairwise F_ST_ values were against population 2 (Table 1). Likewise, subpopulations within each population were also observed in the phylogenetic analysis, providing insight into the relationships between populations. One of the subpopulations of population 3 was genetically related to population 1, indicating that they share a large number of common alleles. The close genetic relationships between populations 1, 3, and 4 suggested that population 2 is the founder population in Senegal. The association of phenotypic traits in the structure analysis of this population will be useful to identify populations with a high frequency of particular traits, and/or to prevent the use of highly genetically related accessions in breeding programs.

### Allelic diversity

The allele frequency distribution for this NPGS Senegal collection revealed that 70% were rare (minor allele frequency <0.05). A high frequency of rare alleles was also observed in the analysis of 137 and 354 sorghum accessions from the NPGS Ethiopia collection [44], and 2,815 maize inbred accessions from the NPGS collection [45]. This large number of rare alleles could be the result of the use of high polymorphism markers for the Senegalese germplasm based on previous genetic diversity studies [29]. The number of alleles in a population provides insight into its genetic diversity. Herein, the allelic richness in Guinea populations 1, 2, and 3 averaged 2.8, 3.6, and 3.5 alleles, respectively, whereas the multi-race population 4 averaged 4.8 alleles (Table 2). Moreover, population size was not correlated with the number of alleles. Populations 2 (n = 29) and 4 (n = 15) comprised 105 and 109 alleles, respectively, whereas populations 1 (n = 7) and 3 (n = 56) comprised 89 and 113 alleles, respectively. To compare the diversity between these four populations, we determined the total number of private alleles in each population. We identified 98 private alleles (44%) that characterized these four populations, of which 98% were rare. Remarkably, populations 2 and 4 had the highest number of private alleles (30 and 37, respectively), which account for 29% and 34% of the total number of alleles, respectively. Populations 1 and 3 had the lowest number of private alleles (11 and 20, respectively). Because population 4 includes multiple sorghum races, its private alleles cannot be used as indicators of genetic diversity. Nevertheless, the private alleles in populations 1, 2, and 3 could be considered signatures of selection in the Guinea-race for the eco-geographical regions or the agricultural system of Senegal. Likewise, allelic richness could be monitored as an indicator of the conservation of its genetic diversity in the NPGS germplasm collection [46].

**Table 2:** Allelic diversity of NPGS Senegal sorghum germplasm populations.

SSR	No. alleles	Allelic richness^1^				H_OBS_^2^	H_EXP_^2^	F_ST_^2^
		Pop. 1	Pop. 2	Pop. 3	Pop. 4			
Xisep310	5	2	3	3	2	0.26	0.33	0.35
mSbCIR223	8	2	3	4	4	0.82	0.60	0.24
mSbCIR240	18	4	1	5	6	0.13	0.56	0.22
mSbCIR262	10	4	3	5	3	0.24	0.59	0.19
mSbCIR283	19	3	5	5	7	0.21	0.75	0.15
mSbCIR286	10	3	3	3	5	0.10	0.54	0.26
mSbCIR306	7	2	2	3	4	0.39	0.57	0.06
mSbCIR329	8	2	2	3	5	0.12	0.40	0.30
Xcup14	10	1	4	2	3	0.10	0.25	0.47
Xcup61	5	2	1	2	3	0.32	0.35	0.32
Xcup63	11	4	4	4	4	0.70	0.69	0.02
Xgap206	16	3	7	3	9	0.18	0.60	0.17
Xisep107	3	1	1	1	3	0.03	0.11	0.19
Xtxp010	10	1	4	3	5	0.06	0.51	0.11
Xtxp015	14	6	5	5	4	0.15	0.73	0.09
Xtxp040	9	2	4	3	5	0.48	0.60	0.16
Xtxp114	3	2	2	2	3	0.21	0.35	0.30
Xtxp265	21	2	8	4	8	0.27	0.69	0.13
Xtxp321	21	6	7	7	7	0.22	0.83	0.09
Xtxp145	13	3	3	2	6	0.22	0.53	0.19

^1^ Allelic richness.

^2^ H_OBS_, H_EXP_, and F_ST_ refer to observed and expected heterozygosity and fixation index of the markers.

### Phenotypic diversity

The NPGS Senegalese collection includes four populations, of which three (populations 1, 2, and 3) have Guinea-race germplasm and the other (population 4) includes multiple sorghum races. The phenotypic characterization of these populations provides insight into their potential use in breeding programs. We characterized these populations with respect to four agronomic traits and two fungal diseases of potential interest to the sorghum breeding community.

The phenotypic diversity of the Senegal germplasm was associated with the observed population structure. Five evaluated traits showed differences between populations (Table 3). FL did not differ between populations (i.e., no photoperiod sensitivity effects during experiments), suggesting that population 3 was selected for taller plants and larger panicles. Seed germination rates were high in population 1; however, this was not sufficient to associate this population with a high frequency of grain mold resistant accessions since is also require to have a superior seed visual ratings. The frequency of anthracnose resistance responses was low in population 4, and the Guinea-race (i.e., populations 1, 2, and 3) had a higher frequency of anthracnose resistance accessions than other sorghum races (i.e. population 4).

**Table 3:** Phenotype analysis of NPGS Senegal sorghum germplasm populations.

Controls	Flowering^1^	Height^2^	Panicle		Grain mold		Anthracnose^7^		
			Length^3^	Diameter^4^	Rating^5^	Germination^6^			
BTx623	72 ± 3	118 ± 11	18.6 ± 3.5	3.1 ± 0.9	3.5 ± 0.5	59 ± 8	S		
RTx430	77 ± 3	113 ± 11	28.8 ± 3.0	4.1 ± 0.8	4.0 ± 0.4	55 ± 6	S		
SC748-5	73 ± 3	105 ± 11	22.0 ± 3.0	2.9 ± 0.8	4.3 ± 0.3	82 ± 5	R		
SC112-14	68 ± 6	140 ± 22	22.3 ± 6.0	4.7 ± 1.6	4.2 ± 0.6	75 ± 11	R		
Sureño	75 ± 4	144 ± 14	14.7 ± 6.0	3.3 ± 1.6	2.4 ± 0.5	87 ± 9	S		
PI 267548	73 ± 3	206 ± 11	30.4 ± 3.0	5.8 ± 0.8	1.7 ± 0.3	92 ± 5	S		
RTx2911	77 ± 4	141 ± 15	26.3 ± 6.0	3.7 ± 1.6	4.1 ± 0.5	60 ± 9	S		
**Senegal germplasm**							**R**	**S**	***X***^***2***^ ***test***
Population 1 (n = 50)	59 ± 1	180 ± 3	27.7 ± 0.8	5.0 ± 0.2	3.3 ± 0.1	89 ± 1	0.60	0.40	0.30
Population 2 (n = 29)	60 ± 1	168 ± 4	27.2 ± 0.9	4.5 ± 0.2	3.0 ± 0.1	82 ± 2	0.48	0.52	0.69
Population 3 (n = 62)	60 ± 1	212 ± 3	33.1 ± 0.7	5.9 ± 0.2	3.0 ± 0.1	87 ± 1	0.52	0.48	0.97
Population 4 (n = 16)	64 ± 1	168 ± 6	23.6 ± 1.5	5.5 ± 0.4	3.4 ± 0.2	80 ± 4	0.11	0.89	0.00
Admixed (n = 10)	61 ± 2	176 ± 7	28.0 ± 1.7	5.1 ± 0.4	3.5 ± 0.2	84 ± 3	0.33	0.67	0.16
**Population means analysis**									
1 vs. 2	n.s.	n.s.	n.s.	n.s.	n.s	***			
1 vs. 3	n.s.	***	***	***	n.s	n.s.			
1 vs. 4	n.s.	n.s.	n.s.	n.s.	n.s.	***			
2 vs. 3	n.s.	***	***	***	n.s.	n.s.			
2 vs. 4	n.s	n.s.	n.s.	n.s.	n.s	n.s.			
3 vs. 4	n.s.	***	***	n.s.	n.s.	***			

Phenotype evaluations were completed at Isabela and Mayaguez, Puerto Rico, during the fall of 2014.

^1^ Flowering time refers to days to 50% flowering of the plot.

^2^ Plant height (cm) refers to the distance from the base of the main stalk to the top of the panicle.

^3^ Panicle length (cm) refers to the distance from the base to the top of the panicle.

^4^ Panicle diameter (cm) refers to the widest region of the panicle.

^5^ Seed mold rating based on a scale of 1–5 where 1–2 are resistant and 3–5 are susceptible.

^6^ Germination rate based on the percent of seeds that germinated after 10 days in flats containing Metro Mix 200 potting medium.

^7^ Anthracnose resistance response where 1–2 are resistant and 3–5 susceptible.

*, **, *** refer to Tukey-Kramer HSD significant differences at p < .05, .01, and .001, respectively.

**Fig 4:**
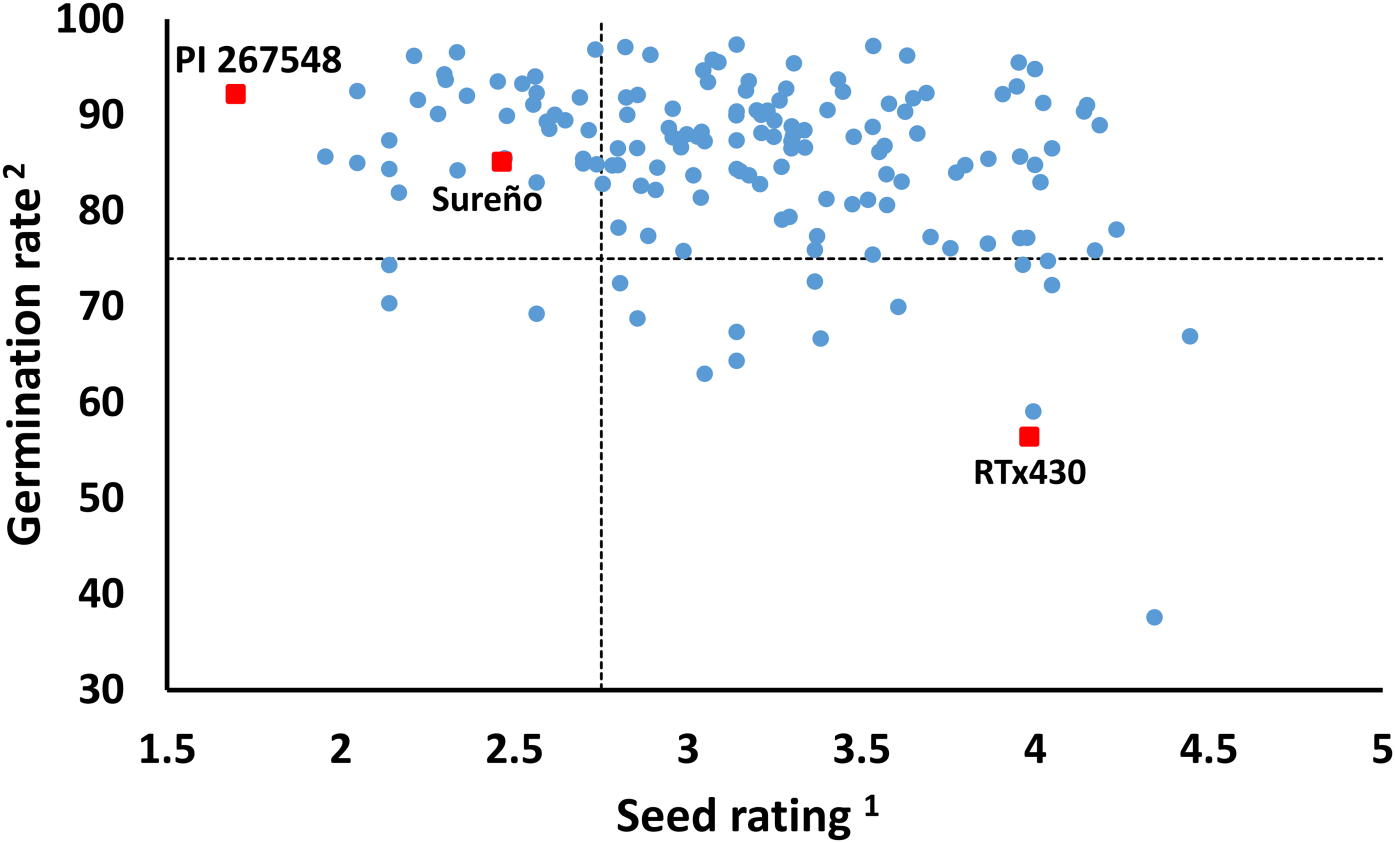
Seed quality distribution of 158 NPGS Senegal sorghum accessions and three references lines. Seed quality evaluations were completed at Isabela and Mayaguez, Puerto Rico, during the fall of 2014. Dashed lines indicate Tukey-Kramer HSD significant differences (*p* < .05) from PI 267548. ^1^ Seed mold rating based on a scale of 1–5 where 1–2 are resistant and 3–5 are susceptible. ^2^ Germination rate based on the percent of seeds that germinated after 10 days in flats containing Metro Mix 200 potting medium.

The Guinea-race is associated with selection favoring resistance against fungal diseases. In the present study, we confirmed that this germplasm is an important source of anthracnose and grain mold resistance (Table 3). More than 50% of the accessions (81) were anthracnose resistant, and they were evenly distributed between populations 1, 2, and 3. The high humidity of West and Central Africa may have driven the evolution towards and selection for anthracnose resistance responses. For instance, the frequency of resistant accessions is low in the Northeastern [Ethiopia (7%), Sudan (24%)] and Southern [Botswana (34%) and Zimbabwe (37%)] regions of Africa [23, 24, 27]. Similarly, the average seed rating and germination in the Senegal germplasm is higher than that observed in Sudan [47] and South Africa [19]. Indeed, the germination rate of most of the Senegal accessions was similar to that of the resistant controls (PI 267548), whereas only 30 had a similar seed rating [<2.74 (Fig 4)]. Therefore, 14 accessions were classified as grain mold resistant (germination >75% and seed rating <2.74) and distributed among populations 1 (2), 2 (3), 3 (8), and 4 (1) (Table 4). Hence, the population structure suggests the presence of up to three different resistance sources for both diseases.

**Table 4:** Seed quality of NPGS Senegal sorghum accessions resistant to grain mold.

**Controls**			**Seeds**		
			**Rating**^2^	**Germination**^3^	
PI 267548			1.70 ± 0.48	92.0 ± 0.1	
Sureño			2.33 ± 0.58	85.0 ± 0.1	
RTx430			4.00 ± 0.71	57.0 ± 0.3	
**NPGS Senegal**	**Population**^1^	**CM**^1^	**Rating**^2^	**Germination**^3^	**Anthracnose**^4^
PI_514391	Pop 1	0.97	2.25 ± 0.50	91.0 ± 0.1	S
PI_514302	Pop 1	0.99	2.40 ± 0.89	94.2 ± 0.0	R
PI_514466	Pop 2	0.99	2.20 ± 0.45	89.2 ± 0.1	R
PI_514453	Pop 2	0.99	2.33 ± 0.58	92.0 ± 0.1	R
PI_514449	Pop 2	0.99	2.50 ± 0.71	93.5 ± 0.0	R
PI_514472	Pop 3	0.79	2.25 ± 0.50	95.3 ± 0.0	R
PI_514473	Pop 3	0.89	2.33 ± 0.52	96.8 ± 0.0	S
PI_514338	Pop 3	0.89	2.50 ± 0.58	89.0 ± 0.1	S
PI_514374	Pop 3	0.97	2.50 ± 0.55	91.7 ± 0.1	R
PI_514420	Pop 3	0.99	2.50 ± 1.05	86.3 ± 0.1	S
PI_514456	Pop 3	0.98	2.50 ± 1.29	91.0 ± 0.0	S
PI_514349	Pop 3	0.98	2.57 ± 0.98	91.4 ± 0.1	R
PI_514380	Pop 3	0.99	2.57 ± 0.53	90.4 ± 0.1	S
PI_514467	Pop 4	0.75	2.17 ± 0.75	83.5 ± 0.2	R

Seed quality evaluations were completed at Isabela and Mayaguez, Puerto Rico, during the fall of 2014.

^1^ NPGS Senegal sorghum populations based on the STRUCTURE analysis of 221 SSR alleles, and accession coefficient of membership.

^2^ Seed mold rating based on a scale of 1–5 where 1–2 are resistant and 3–5 are susceptible.

^3^ Germination rate based on the percent of seeds that germinated after 10 days in flats containing Metro Mix 200 potting medium.

^4^ Anthracnose resistance response at Isabela and Mayaguez, Puerto Rico, where S and R refer to susceptible and resistant, respectively.

## Discussion

The Senegalese collection represents a minor portion of the NPGS sorghum germplasm that can be rapidly phenotyped for important traits in agriculture. Although germplasm from this region is considered an important source of disease-resistance genes, the Senegal NPGS germplasm collection has not been used in sorghum breeding programs. Therefore, the genetic characterization presented herein will be of value to identify and make adequate use of the most genetically valuable germplasm for breeders and geneticists worldwide. The use of the most genetically diverse accessions reduces the labor required to screen highly genetically related accessions (i.e., identical by descent) and increases the potential to identify agronomically superior accessions and new sources of disease resistance for the development of new cultivars in breeding programs.

Population structure analysis within Guinea race germplasm can be used to identify accessions with high frequency of desired agronomic traits. The high frequency of taller plants within Population 3 suggest these accessions could enclose genes selected for high biomass not linked to flowering time. In fact, sorghum is a dual purpose crop in the subsistence agricultural system of Senegal; where the grain are used for human and animal consumption, while the stalks are employed to feed animals after harvesting. The high frequency of disease resistance in the Senegalese germplasm could not be linked to a bountiful presence of disease-resistance genes. The population structure of the collection suggests the presence of limited sources of resistance that were fixed in this germplasm. The integration of accessions with resistance to multiple diseases that belong to different populations (Table 4) is the most adequate strategy for the success of breeding programs. In this regard, the identification of genomic regions and molecular markers associated with these sources of resistance may contribute to accelerate the development of new varieties. However, the polygenic inheritance and low heritability of the grain mold resistance response [48, 49] are not suitable for genomic dissection by association mapping approaches. Instead, the development and use of multiple bi-parental populations with the most genetically diverse accessions from each population is the most adequate approach. Similarly, the large number of NPGS accessions from West and Central Africa [Senegal (357), Gambia (67), Sierra Leone (27), Liberia (4), Cote d’Ivoire (2), Ghana (54), Togo (565), Burkina Faso (354), Nigeria (577), Cameroon (250), and Central African Republic (8)] should be reduced into a diversity panel for large-scale phenotypic and genotypic analyses. This type of germplasm resource will help identify multiple disease-resistance sources and encourage their inclusion in sorghum breeding programs.

The introgression of genetically diverse and disease resistant Senegalese accessions into sorghum breeding programs requires the modification of multiple traits. First, the anthracnose and grain mold resistance accessions identified herein belong to the Guinea-race; therefore, its panicle shape does not resemble that of the standard commercial-type sorghum. Second, the flowering time of these tropical exotic accessions must be converted to adapt to temperate grain production regions, as panicle characteristics play an important role in resistance responses [50]. Nevertheless, previous studies based on the intercross of Guinea × Caudatum showed that grain quality (Guinea) and productivity (Caudatum) can be combined in recombinant inbred lines [51]. Therefore, breeding schemes that include temperate climate-adapted germplasm susceptible to grain mold with a good general combining ability [e.g., RTx 430; [52]] could be used to develop new resistant varieties. However, the fixation of temperate-adapted alleles must be delayed to late generations to increase the number of recombination events and the potential to produce a valuable breeding germplasm.

The large size of the NPGS germplasm collection requires the identification of valuable germplasm for phenotyping and its conservation. The genetic differences between Senegal populations (F_ST_ >15) suggests that the establishment of a small representative core set is the best strategy to avoid the screening of genetically redundant germplasm and make better use of field resources. Indeed, the phenotypic evaluation of a subset of samples from each population is adequate to identify sources of disease resistance in the collection. In this regard, a GBS analysis at low coverage (1×) for the whole Senegal collection is appropriate and cost effective to establish a precise core set. Moreover, this genotyping information could be used to determine the genetic relationship of the NPGS Senegalese germplasm with accessions present in the SAP [9], the NPGS Ethiopia collection [11], and other germplasm collections worldwide [10]. The adequate use of the NPGS Senegal collection can broaden the genetic base of sorghum breeding programs.

## Conclusion

The NPGS Senegal sorghum germplasm collection is a highly genetically diverse germplasm composed of four populations. The high frequency of anthracnose and grain mold resistant accessions indicates that these germplasm collections include important genes for breeding programs. Nevertheless, its well-defined population structure indicates the presence of a limited number of resistance sources. This genetic characterization provides sorghum breeders with the knowledge needed to optimize the use of this germplasm for the development of new sorghum cultivars.

## Supporting information

S1 File Senegal Supporting Information.xml. (XLSX)
